# Designing Paper-Based Immunoassays for Biomedical Applications

**DOI:** 10.3390/s19030554

**Published:** 2019-01-29

**Authors:** Delyan R. Hristov, Cristina Rodriguez-Quijada, Jose Gomez-Marquez, Kimberly Hamad-Schifferli

**Affiliations:** 1 Department of Engineering, University of Massachusetts, Boston, MA 02125, USA; Delyan.Hristov@umb.edu (D.R.H.); Cristina.Rodrigue002@umb.edu (C.R.-Q.); 2 Little Devices Lab, Massachusetts Institute of Technology, Cambridge, MA 02139, USA; jfgm@mit.edu

**Keywords:** paper-based immunoassays, lateral flow assay, biological sensors, dipstick assay, rapid diagnostics

## Abstract

Paper-based sensors and assays have been highly attractive for numerous biological applications, including rapid diagnostics and assays for disease detection, food safety, and clinical care. In particular, the paper immunoassay has helped drive many applications in global health due to its low cost and simplicity of operation. This review is aimed at examining the fundamentals of the technology, as well as different implementations of paper-based assays and discuss novel strategies for improving their sensitivity, performance, or enabling new capabilities. These innovations can be categorized into using unique nanoparticle materials and structures for detection via different techniques, novel biological species for recognizing biomarkers, or innovative device design and/or architecture.

## 1. Introduction

Diagnostics are critical for healthcare to confirm or help diagnose a patient. Typically diagnostics are lab-based, often requiring skilled personnel and can be labor intensive. This increases the time and cost of diagnosis and limits their accessibility. The problem is accentuated in more rural communities and in middle and lower income countries where point of care (POC) facilities are limited in equipment and resources to train personnel. This can present a serious challenge for local and lower income healthcare systems as doctors lose faith in pathological evidence and fall back on presumptive diagnosis [1]. 

For example, both dengue and Zika viruses inhabit tropical climates and are transmitted by the *Aedes Aegypti and Aedes albopictus* mosquito. They have similar early stage symptoms, muscle and joint pain, rashes, headaches, fever and other flu-like symptoms, but treatment for both are very different [2]. While for most adults, infection with Zika does not require hospitalization, whereas dengue is more severe leading, to hospitalization for ~100 in 100,000 cases, or death in ~5 in 100,000 cases [3,4]. Furthermore, early stage symptoms of both viral infections can be mistaken other infections, such as chikungunya [5] and Spondweni viruses [6]. While Zika can be mild for adults, there is a confirmed causal relationship between women exposed during pregnancy and microcephaly in the child [7,8]. Differentiating between these and many other diseases is crucial for patient health outcomes, disease surveillance, and emergency preparedness, but often difficult [9]. 

It is estimated that the global biosensor market will grow by over 70% by 2022 from 15.6 billion USD in 2015 to over 27 billion USD [10,11,12]. The majority of the market share (66% in 2013 [10]) is in medical diagnostics, particularly in diabetes, cancer and infectious disease detection. There has been a steep increase in the number of developed diagnostics tools, devices and procedures [13,14]. Advancements in technology and the push for new, low cost, user-friendly POC devices have been cited as the main drivers [10,12]. The World Health Organization (WHO) has established guidelines for diagnostics in the developing world, which are that they must be Affordable, Sensitive, Specific, User Friendly, Rapid and Robust, Equipment Free, Deliverable (ASSURED). The ASSURED criteria are difficult to fully meet, but paper-based assays have the potential to meet most of these requirements [15]. Presently the most commonly used diagnostics are laboratory based techniques, such as PCR [16], enzyme-linked immunosorbent assay (ELISA) [17,18], Western blot [17,18] and cell culture assays. However, paper-based assays are an important technology that is growing.

### 1.1. Paper-based Immunoassays

Paper-based assays are paper strips which can test for the presence of a biomarker in a biological fluid. Most paper-based assays are immunoassays, i.e., they use antibodies to bind to the biomarker of interest. They can be read out by eye, which is due to the use of gold nanoparticle (NP)-antibody conjugates that cause a strong color to appear at a test line. 

Though variations exist in their most basic form paper-based assays consist of a nitrocellulose or glass fiber substrate; NPs functionalized with antibodies, or immunoprobes, and a capture antibody printed onto the substrate. Due to the small number of components, lack of moving parts, and minimal quantities of reagents, single assays are low cost and easy to mass produce [19,20].

**Figure 1:**
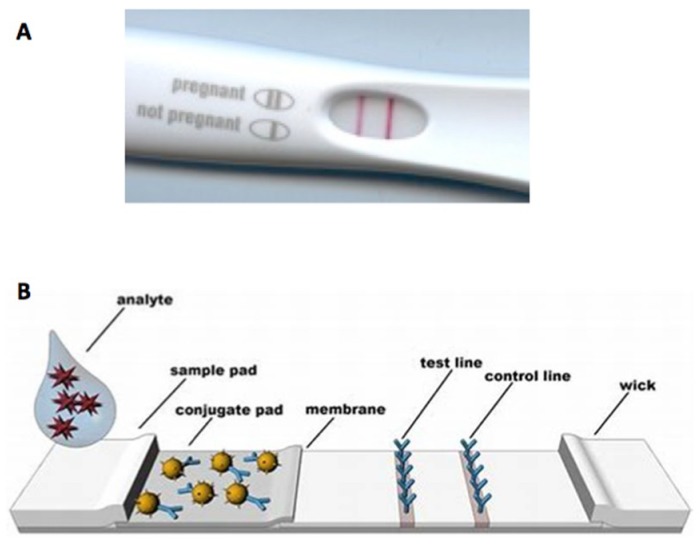
(**A**) Pregnancy test and (**B**) structure of a lateral flow assay. From Miočević, O. et al. [24].

A wide range of molecules can be bound to NPs to form the probes used in paper-based assays, to enable numerous possible targets and applications. Furthermore, multiple probes functionalized with different molecules can be used to simultaneously test for a number of targets [20,21,22]. Device design geometry can be augmented to suit use-specific requirements [19,23]. Paper-based assays do not require power or additional reagents to run, further increasing applicability and reducing costs. A single assay can be complete within minutes. Furthermore, test results can be easily read and understood by non-specialists (Figure 1). The biggest advantages of paper-based assays are their ease of use, portability and low cost. Due to the wide applicability of bioassays and broad background of end users, and these assays can provide an understandable yes/no diagnosis. 

As a result, paper-based assays are considered a critical POC device with the potential to expedite testing times, reduce costs, and enable clinicians to make faster treatment decisions thus improving patient healthcare outcomes [25]. Though paper assays have been successful in many areas, the field is multidisciplinary and complex, and as a result it can be difficult to identify key gaps. This review aims to provide an understanding of the fundamentals of paper-based assays and introduce the reader to various related contemporary ideas and challenges. We first discuss the fundamentals and define core concepts, such as what a paper-based assay is, fluid flow in paper matrices, and common nanoparticle readouts used. This is followed by a section on challenges designed to present the reader with a set of common pitfalls encountered when designing a new paper-based assay. 

### 1.2. Commercial Applications

The simplicity of use for paper-based assays has made them a potentially transformative technology in a variety of fields. In addition to the most commonly used assay, the over-the-counter pregnancy test, numerous paper-based assays are commercially available (mostly lateral flow type systems) or are in various stages of development (mostly newer designs which include patterning on paper or enhanced signal readouts) for agriculture [26,27,28], environmental protection [29,30], poison [31,32,33], antibiotics [34], infectious disease agents [35,36], noninfectious diseases [37], and many others. In agriculture, tests can be used for compliance to regulatory adherence [27] in soil and water quality analysis [30]. 

### 1.3. Disadvantages

While the simple design and use of paper-based assays is their most attractive feature, it also presents a number of challenges and disadvantages. There are a number of reviews which perform a cost-benefit analysis of paper-based assays [38,39], including strengths, weaknesses, opportunities and threats (SWOT) analysis [35,40]. 

**Qualitative instead of quantitative readouts** [35,41]. The POC format often allows only a qualitative or semi-quantitative result. Some have attempted to resolve this by introducing cell phone attachments which allow for quantification of the test line intensity and thus more accurate result analysis [42].

**Low sensitivity** [43]. While paper assays have benefits of a simple test methodology, do not require instrumentation, have a simple readout and need low sample volumes, these can reduce assay sensitivity. Visual readout means that target biomarkers must be present at a sufficient concentration. Low sensitivity can be ameliorated by signal enhancement techniques [44], improving test design [45] changing the readout type, or the use of instrumentation. 

**Test variability.** Variability of paper-based assays can be high, resulting from complex samples, environmental factors, such as temperature and humidity, paper porosity, and probe instability [38].

**Single use, not high throughput** [35]. Paper-based assays are often by design single use devices and thus not meant for analysis of multiple samples. 

## 2. Components of Paper-Based Assays

Understanding the fundamental principles and controls has enabled researchers to develop a wide range of applications of paper-based assays, such as incorporation of novel probes, strategies for increasing sensitivity, and complex multilevel paper analytical devices [40]. Here we review the basic properties of paper-based tests. 

### 2.1. Paper-Based Assay Formats: Dipstick and Lateral Flow Configurations

**Figure 2:**
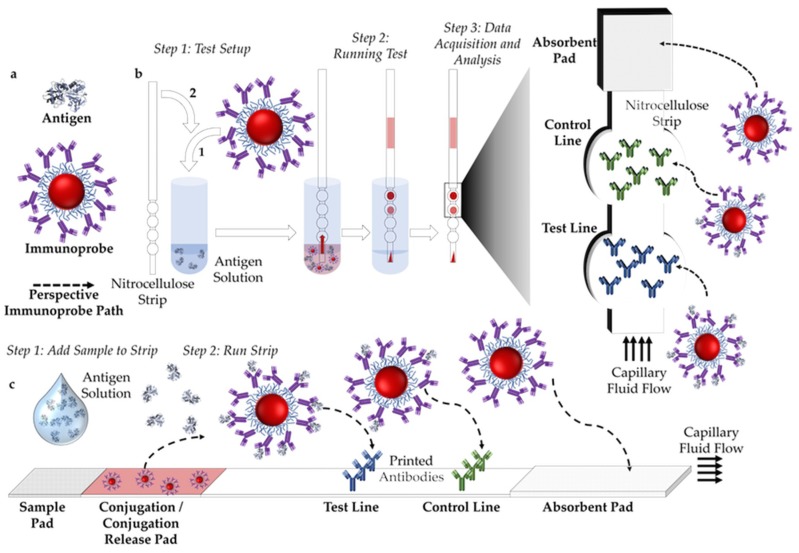
(**a**) Figure legend. (**b**) Schematic representation of a test run and result readout of a dipstick assay configuration. (**c**) Schematic representation of a test run and result readout of a lateral flow assay (LFA) configuration.

Paper-based assays are comprised of a nitrocellulose substrate (or strip), an immunoprobe (often gold NP based) and a sample solution (Figure 2a). Antibodies are printed onto the test strip, one which recognizes the biomarker-antigen or antibody- (on the test line, blue in Figure 2b), and another which recognizes the antibody bound to the NP (on the control line, green in Figure 2b). Readout is based on the presented pattern, where the test line indicates the presence/absence of the biomarker and the control line indicates that the test has run properly. 

There are two main assay formats, either dipstick or lateral flow (Figure 2b,c). For lateral flow assays (LFAs), the strip consists of a sample pad, conjugation pad, test area and an absorbent pad. After the sample is deposited onto the pad it runs through the paper due to capillary flow. Pre-dried immunoprobes on the conjugation pad are rehydrated and start diffusing in the solution and bind with the antigen (Figure 2c). An absorbent pad attached to the nitrocellulose strip acts as a fluid sink, providing the driving force for capillarity. In a dipstick assay there are no sample or conjugation pads, where the immunoprobes are mixed with the sample and the bottom of the strip is immersed in this solution (Figure 2b). 

**Figure 3:**
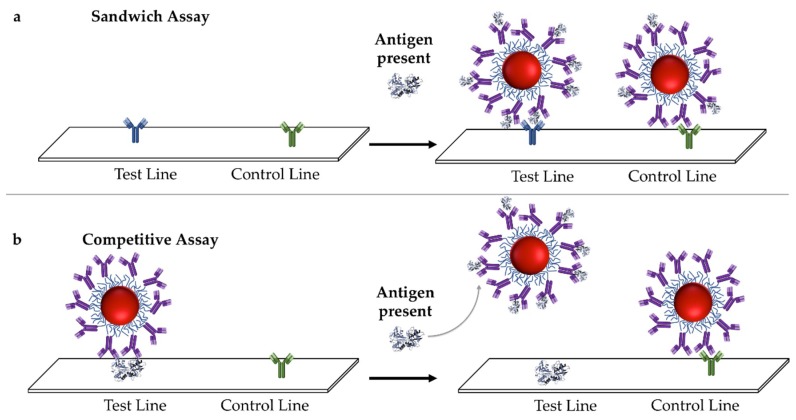
Signal origin in an LFA using the (**a**) sandwich format, where an antibody specific for the antigen is immobilized on the test line, and (**b**) competitive format, where an antigen is immobilized on the test in and pre-bound with the nanoparticle (NP)-antibody conjugate. If the antigen is present in the fluid, the NP-antibody conjugate is displaced from the test line, resulting in a loss of color from the test line.

There are two binding configurations for immunoassays. A sandwich configuration is when the presence of the target antigen results in simultaneous binding to the immunoprobe and capture antibodies (Figure 3a) [38,41,46,47], while the control antibody binds to the probe only. A positive recognition event is expressed as the appearance of a line in the test area (Figure 3a). If the target is too small to bind two antibodies simultaneously, or has epitopes for that inhibit one another, a competitive format can be used [38,41,46,47,48]. A pre-bound immunoprobe is present at the test line, and when the target molecule is present it displaces the immunoprobe, resulting in the disappearance of color from the test line (Figure 3b). 

### 2.2. Paper Assay Components

A paper assay diagnostic is composed of several components:

#### 2.2.1. Paper Substrate

Paper is the most commonly used substrate for NP immunoassays [19,20,41,49]. Because it is porous, liquid can flow through it without external pumping [19]. It is biologically compatible and does not strongly interact with biomolecules and immunoprobes within its matrix, but can be chemically modified if required [49]. Paper also has a large surface area which allows the immobilization of a large number of sensor molecules [49]. It is white which leads to better contrast.

Paper is thin, light weight, flexible, durable, and easy to produce, store and manipulate. It is available in a wide variety of sizes, thicknesses and shapes which allows for a measure of control over test running conditions [49]. Furthermore, paper has a long history of application in chemistry, so there is an established production and supply infrastructure in place. This makes it widely available in a variety of pore size, types and chemical modifications (e.g., nitrocellulose) [20,49,50]. Instruments to shape paper, such as laser cutters, are affordable user friendly [20]. Finally, it can be produced and disposed of in biologically friendly and safe ways [19]. 

There are several relevant parameters of the paper substrate. Porosity, or void or pore volume fraction, is a measure of the air filled and solid fractions of paper. It is defined as $\varphi =\frac{V_{V}}{V_{T}}\times 100$, where $V_{V}$ is void volume and $V_{T}$ the total volume of the paper [51]. Alternatively it is defined using the density of the solid component wood fiber used to make the paper and the density of the paper itself as $\varphi =1-\frac{\rho_{paper}}{\rho_{solid\;wood\;fiber}}$, where $\rho_{paper}$ and $\rho_{solid\;wood\;fiber}$ are the density of the wood fiber and paper, respectively [49]. Porosity can be broken down into pore size, the average diameter of pores in the paper, and pore size distribution, the distribution of pore sizes in the medium. 

Measurement of the three parameters requires multiple methods, including mercury porosimetry and gas adsorption [51,52]. Often pore sizes provided by manufacturers are a bulk average [53].

#### 2.2.2. Reporters

Reporters indicate the presence of the target antigen, and are linked to the antibody or other binding moiety. NPs are often used as reporters, with gold NPs the most prevalent as they have a high signal to noise ratio for visual readouts. NPs of other materials have been used, such as dye-infused cellulose particles [54], and quantum dots can be used to provide a fluorescent signal [55].

**Figure 4:**
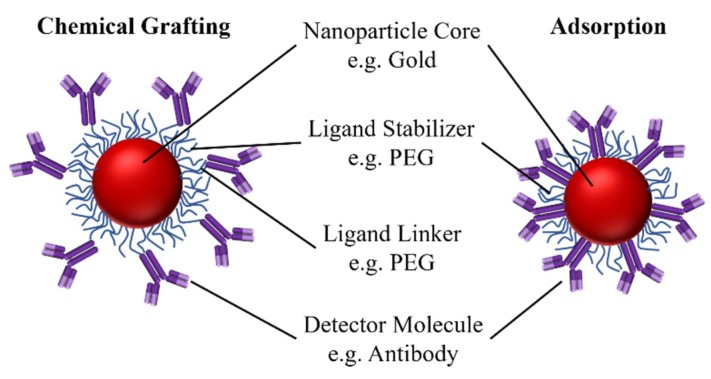
A schematic of immunoprobes where antibodies are attached to the NP core covalently, i.e., through chemical grafting, or physicochemically, i.e., through adsorption.

The immunoprobe is comprised of a NP core, a surface coating ligand to stabilize the NP or enable chemical grafting, and a biomolecule, such as an antibody that binds specifically to the target biomarker (Figure 4). Chemical grafting and physical adsorption are the two strategies used to bind the active molecule to the core.

NPs offer a high surface area and can be attached to many different binding species (antibodies, peptides, DNA, etc.). They are also attractive due to their size-tunable properties, ability to encapsulate and protect many fluorescent entities, and general structural robustness [14].

Multiple factors need to be considered when designing an immunoprobe, including stability in relevant media with respect to both aggregation and dissolution, biomolecule adsorption on its surface, denaturation and accessibility the surface bound antibody, and its overall ability to bind the antigen. In this context the minimum requirements for an immunoprobe to be applicable in paper-based assays:

- Surface bound antibodies are able to bind antigens, i.e., their epitope is still accessible and functional.
- Immunoprobes interact with the environment in a way which does not impede the antibody’s ability to bind the antigen, e.g., particles do not aggregate in relevant conditions.
- Immunoprobes interact with the paper medium weakly and without undesirable precipitation, and can diffuse through the paper strip while producing minimal background.

Due to the large number of antigen targets and NP types in paper assays, there is an enormous number of immunoprobe types in literature. In a 2017 review Ferka Z. et. al. identified 3300 relevant papers published between 2012 to 2016 [47]. 

Reporters can also be chemical reagents used, including pH indicators, dyes, antibodies, and enzymes [43]. Small molecules are sometimes nonideal due to the low signal-to-noise of a single molecule. Furthermore it is a common practice in paper production to add fluorophores to make the product look whiter, which could cause a high background [49]. 

#### 2.2.3. Antibodies that Bind to the Target

When creating a diagnostic for a pathogen of an infectious disease, one must choose a target biomarker which must have clinical relevance and be present at high enough concentrations in the fluid of interest. For viral or bacterial infections, one can detect the virus or bacteria present in the patient, usually via the envelope proteins or the genetic material of the pathogen. Alternatively, the immune response of the patient can be used for diagnosis, where the antibodies generated in response to the infection are detected [54,56]. In each case, antibodies specific for the target biomarker are attached to the reporter and also immobilized on the strip.

Antibodies are proteins produced by a host in response to an antigen, and thus can bind to it with specificity. Antibody-antigen interactions are characterized by high affinity and specificity, which makes them an attractive binding species. They are a family of proteins which are referred to as immunoglobulins (Igs) which are produced by differentiated B cells. There are five primary classes of Igs: (i) IgA is the second most common in human serum and is most prevalent in human secretions, such as saliva, tears, etc.; (ii) IgD is found in very low concentrations in human serum and the least understood; (iii) IgE is least abundant in human serum; (iv) IgG is the most common in human serum; (v) IgM is the third most common in human serum, but is expressed at the early stages of infection, i.e., on the surface of immature and mature B cells. Antibodies can be either monoclonal or polyclonal, where monoclonal antibodies react with a single, defined site on the antigen (an epitope) while polyclonal antibodies react to multiple sites on the antigen.

Detecting the antigen vs. the antibody depends on disease pathology. For flaviviruses, such as dengue, the antigen nonstructural protein 1 (NS1) serves as a good biomarker, because it is secreted early after infection (within ~1–2 days) when patient symptoms are still non-specific Also, dengue NS1 is present at high concentrations in the blood [56]. Lyme is another good example of this dynamic. Presently the Center for Disease Control and Prevention (CDC) recommends a two tier (ELISA and Western blot) serologic analysis. Due to the nature of the cause (*Borrelia Burgdorferi*) most available tests are for IgG/IgM. This strategy is characterized by high specificity (>98%) and reasonable but variable sensitivity (30–100%) depending on proper application, type of test, and disease stage [18,57,58,59]. However, as detection relies on the host immune response, it is ineffective until a few weeks after the infection [18,60]. *Borrelia* can affect the immune system and in particular IgG production [61,62,63,64] and less so for early IgM production. Furthermore, anti-*Borrelia* IgG can persist for months to years after the infection has cleared, or could be the result of a Lyme vaccine, thus resulting in a false positive [17,18,58]. However, especially at late stages *Borrelia* is mostly found in low numbers in collagenous tissue and thus difficult to detect directly. An assay for a Lyme antigen as opposed to Lyme antibodies would lead to faster and possibly more accurate diagnosis, and potentially lower medical costs and improved patient health outcomes.

For cases when nucleic acids are the target biomarkers, such as detection of viruses or bacteria themselves, or in the detection of siRNA, the capture molecule can be a complementary nucleic acid [39,44,56,65]. This often occurs downstream of PCR or another amplification technique in order to amplify the target nucleic acid. Design of capture nucleic acids can be easily tailored to increase affinity by extending the complementary sequence.

## 3. Metrics for Paper Assay Performance

### 3.1. Description of Target-Antibody Binding

For binding events it is useful to quantify the affinity of the antigen-antibody interaction, as it is occurring at the test line and is responsible for the signal. Because the antibodies are tethered to the nitrocellulose or a NP, the antibody-antigen affinity may not be the same for the free species in solution. Towards this end, a modified Langmuir model can be used to qualitatively evaluate binding affinity [66].

**Figure 5:**
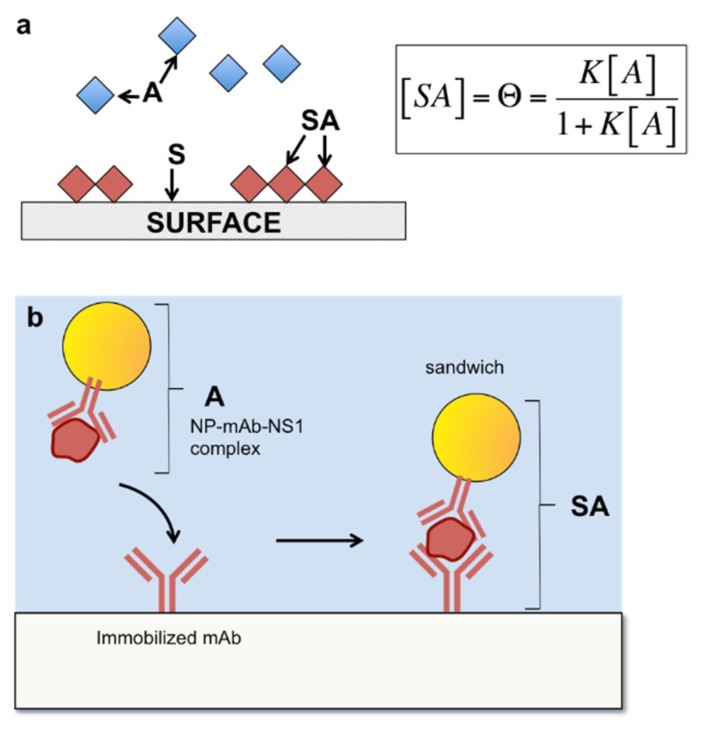
Langmuir binding isotherm adapted to describe the binding event in a sandwich immunoassay. (**a**) Schematic of the free species A adsorbing onto a surface S, forming a surface bound species, SA. (**b**) Analogous schematic with the NP-Ab conjugate bound to NS1 binding to surface bound antibody on the test line. From Reference [66].

The model utilizes the surface adsorption model where the free species is the NP-Ab/antigen complex, and describes its binding to the immobilized Ab on the test line, or successful sandwich immunoassay formation (Figure 5). A free species *A* can bind to a surface species *S*, to form a surface immobilized species, *SA* (Equation (1))

(1) S+A↔SA

**Figure 6:**
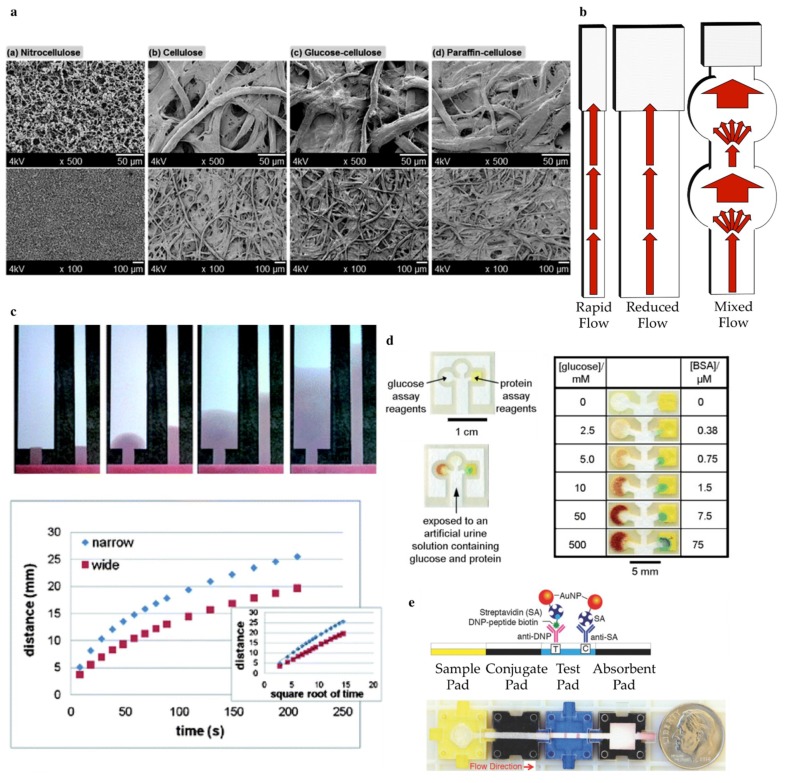
(**a**) Scanning electron microscopy images of nitrocellulose and cellulose from Reference [83]. (**b**) Schematic representation of fluid flow rate in strips with variant geometries compared to (**c**) an experimental example obtained from Reference [80]. (**d**) Example printed µPAD geometry theoretical representation and experimental data of fluid flow over time obtained from Reference [23]. (**e**) The assembled Ampli assay from [85].

The fraction of occupied sites, Θ, can be measured as a function of the concentration of A to obtain an expression containing *K_D_*, the equilibrium dissociation constant (Equation (2)):

(2) $$\Theta =\frac{\mathrm{nK}\left[A\right]}{1+K\left[A\right]},$$

where the equilibrium dissociation constant, *K_D_*, is (Equation (3)):

(3) $$K_{D}=\;\frac{\left[S\right]\left[A\right]}{\left[\mathrm{SA}\right]},$$

which describes the affinity of *A* for *S*. Here, the Langmuir surface model describes the second binding event, so *A* is taken to represent the NP-Ab/antigen complex and *S* is the immobilized antibody, and the sandwich immunoassay represented by *SA* (Figure 6b). The concentration of *SA* is proportional to the test line intensity, which is proportional to the number of NPs at the test line due to target binding. 

It is important to note that the model assumes that the surface bound species *SA* are independent and do not influence binding of adjacent sites, and that surface binding can result only in a monolayer and equilibrium binding conditions [67]. The binding affinity constant is an effective binding constant, *K_D_^eff^*, as the binding events are lumped together as binding of the NP-antibody/antigen complex to the immobilized antibody. Nevertheless, *K_D_^eff^* can still be used to compare how binding affinity changes under different conditions, such as NP surface chemistry, NP properties, and running conditions. 

### 3.2. Sensitivity and Specificity

Paper-based assay performance is assessed by their sensitivity and specificity [68]. Sensitivity is a measure of the ability of the assay to correctly detect the target, and calculated using Equation (4) (Table 1) as a percentage. An important element of sensitivity is the limit of detection (LOD) [47,69,70] defined as the concentration at which there is a signal that is significantly higher than the background. Typically this is defined as 3X the standard deviation of the blank above the baseline [71]. LOD depends on the antibody-antigen affinity and immunoprobe physical properties (optical absorption for colorimetric readouts). Other factors include paper substrate properties, number and state of printed detector molecules the substrate, immunoprobe stability, ability of both printed and immunoprobe bound detector molecules to the target, readout method, competition with free target molecules [36,72].

Specificity is a measure of the likelihood of the test to give a false positive (Table 1, Equation (5)). For example, if a test gives a positive result for a sample which does not contain the target molecule half of the time, it has a specificity of 50%. Similarly to sensitivity and LOD, specificity depends on a wide range of factors, but especially cross reactivity and nonspecific binding either of molecules to the immunoprobe surface or of the immunoprobe on the paper.

## 4. Factors Impacting Paper Assay Performance

### 4.1. Fluidic Properties of The Paper Strip

The rate at which the fluid flows through the strip can influence assay performance [74,75]. Slowing down flow can improve antigen binding with the immobilized antibodies and immunoprobe. Control of flow can be used to improve reagent mixing or even achieve multiple reactions in a sequential and timed order [45,76,77,78,79]. Typically, in the construction of a paper-based assay, design of the strip can be used to control flow through the following means:

**Flow rate control through strip geometry.** Widening the strip results in a slower flow rate and thus longer time for reactions to occur, but also increases sample and reagent volume requirements and test time [74]. In extreme cases it may result in uneven flow [49,74,76,80]. Increasing the strip length inversely impacts fluid flow [80,81] and can increase the probability of a successful antigen binding, but may also increase the probability of NPs getting stuck in the paper matrix. Hence there is a maximal desirable channel width and length for a given use. Some [76,80,82] have developed a mixed geometry with narrow channel regions and wide control and test regions (Figure 6b,c). This allows for slower flow as the sample passes over the printed antibodies to increase the success rate of binding. 

**Flow rate control through paper porosity and morphology**. Paper is composed of cellulose fibres, which are hollow tubes with an average length of 1.5 mm, width of 20 µm and wall thickness of 2 µm [49]. The paper pore size distribution is largely dependent on the production method and post production treatment [11,75]. Nitrocellulose is predominantly used compared to cellulose [75], where the hydroxyl groups in cellulose are replaced with nitride groups. Consequently, the pore size of the paper reduced from an average of tens to hundreds of µm to hundreds of nm, its surface becomes more hydrophobic and its structure becomes amorphous [20,43,83] (Figure 6a). The size and morphology of nitrocellulose pores are dependent on casting conditions, such as water content and additives [75].

Pore size impacts not only fluid flow but also assay dynamics [84]. Small pores may cause immunoprobes to get stuck in the matrix, while larger pores may result in an increase of test surface area where a binding event can occur. Furthermore, paper is anisotropic [49], where the preferential fiber orientation can impact fluid flow [19].

### 4.2. Sample Preparation

Paper-based immunoassays usually require the addition of a pre-treated biological sample (blood, urine, saliva, and others) in order to flow properly throughout the assay. Although a biological matrix has proven to improve the performance of LFAs [66], typically blood-based tests require a sample preparation step to remove red blood cells that can interact non-specifically with the NP probes and result in false positives. This is conducted either by a pretreatment of the fresh blood with anticoagulant molecules (e.g., heparin) and further centrifugation of blood cells (plasma) or by the centrifugation of blood after coagulation to eliminate not only blood cells but also coagulated material (serum). In an attempt to eliminate this extra step which requires equipment that may not be accessible in certain POC facilities, some paper-based assays have included a paper fiber matrix that retains blood cells [86]. The eluted solution (plasma) flows through capillarity downstream this step and functions as a typical LF test.

## 5. Novel Modifications of Paper-Based Assays

While paper-based assays have the potential to transform POC diagnostics, there are still many approaches being studied to improve their performance. Presently, multiple strategies are in development to address this issue varying from improving and understanding immunoprobe design and test modification, to implementing smartphone and other affordable instrumentation for more advanced readout (Table 2).

### 5.1. Modifications and Improvements of Paper-Based Assays

#### 5.1.1. Paper Enhancements

Paper-based immunoassays are analogues of ELISA reactions. Running a bulk ELISA requires a stepwise protocol of mixing of solutions in vessels, where reactions must occur sequential after prescribed time periods. The kinetics of the reactions influences the assay sensitivity, so the ability to tune the reaction timings can be used to optimize the assay. Because of this, the ability to introduce timing mechanisms in a paper assay enables the ability to tune and optimize the sensitivity of the assay. There are many different ways in which paper assays can be modified for their fluidic structures to introduce mechanisms for timing of the reaction to enhance the sensitivity, leverage the kinetics of the reactions.

**Flow rate control through additives.** For some applications, e.g., simultaneous detection of multiple antigens, the use of a single sheet of paper is preferred. Multiple centers and/or fine control over liquid flow can be achieved through printing of different products onto the paper substrate. This could be due to the formation of impassable hydrophobic barriers, such as those made from wax [23,43,74,87,88,89] or dissolvable materials, such as dried sugar lines, designed to slow fluid flow [80,90] (Figure 6d).

The use of hydrophobic barriers has been explored for preventing cross-contamination between adjacent reagent areas. Patterning of wax, silicones, paraffin, and other materials has been used to separate and guide fluids in a paper strip, facilitating multiplexing. Wax patterning has been popular as it can be achieved by many different approaches, such as screen printing, dipping, and dripping. Commercially based wax printers have been leveraged to facilitate patterning of structures, but recently these systems have been discontinued,

When reactions necessary for an immunoassay are run on a lateral flow strip, it is similar to a single pot reaction. Control of the sequence of events is not possible as the geometry of the strip is fixed, and usually single lane. However, the introduction of architectures to the paper can result in novel control methods. Two-dimensional paper networks (2DPN) are strategically shaped strips, with incorporated structures that act as valves to enable timing of flow. Fluidic disconnects can be used control fluid arrival times and shut off times [90]. This can result in the sequential delivery of reagents to optimize reaction efficiency and thus assay sensitivity [80].

On the fly customization can be achieved by employing a LEGO-block like system [85]. This is a recently developed strategy called *Asynchronous Modular Paperfluidic Linear Instrument-free* (Ampli), which involves the use of blocks to construct a custom flow arrangement on demand (Figure 6e). Traditional LFAs are constructed of a sample pad, conjugate pad, test area, and wick that are fused together (Figure 1) and the end user receives them as a monolithic device. However, modularizing the device allows the user to vary reaction timing, swapping out of different test areas and reagents, and introduce a desired degree of multiplexing. In an Ampli set, each of the components of the LFA is put on a modular block, and blocks can be easily snapped together in desired configurations to form different numbers of branches for multiplexing, test lane lengths, and incorporate different elements, such as different assays. In addition, Ampli blocks can be dynamically assembled and disassembled, enabling timing of the reagents to maximize signal, or back and forth flow to increase immunoassay test line intensity (Figure 6e).

#### 5.1.2. NP Enhancements

Readout of LFAs is preferably achieved by visualization of the test area due to the accumulation of the NPs, but this can have a limited sensitivity as readout is by eye. Because nanomaterials have many unique size and material dependent properties, different types of probes have been used to enhance the signal intensity in paper-based assays. Increased sensitivity can increase the accuracy of diagnostics or result in earlier detection of diseases. In addition, the unique properties of nanomaterials can be used to introduce new capabilities to paper-based tests, as the materials for the NP probe are broad [14,26,40,41,46,47,70,91,92,93].

**Enhanced optical**. A major drawback of colorimetric readouts is their relatively high LOD. Post processing techniques can lower the LOD of optical readout systems. Test line intensity can be increased by silver staining of the lines lines or isotachaphoresis [94]. Other approaches using NP aggregation [44], secondary probe binding [95,96,97], probe growth [98,99,100], can also increase signal. In addition, the colorimetric change of gold NPs when they aggregate has been leveraged to generate assays that change color from red to blue upon analyte binding [65]. 

**Figure 7:**
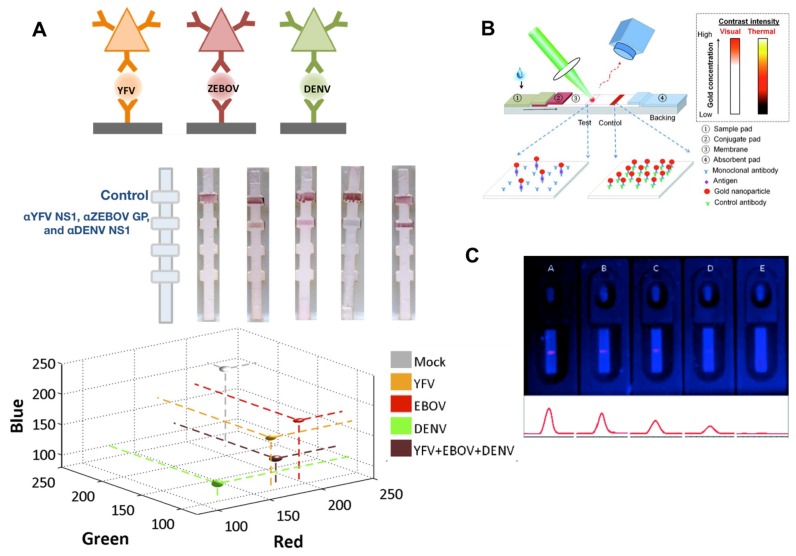
Examples of NP enhancements of LFAs. (**A**) multicolor silver NPs enable multiplexed readout of yellow fever virus, Ebola virus, and dengue virus biomarkers [21]. (**B**) Photothermal enhancement of lateral flow test lines can increase signal contrast [107]. (**C**) Quantum dots can provide a fluorescent readout of LFAs [109]. Reprinted with permission from (Li et al., Analytical Chemistry. Copyright (2010) American Chemical Society."

Fluorescence readouts can increase sensitivity and lower LOD (Figure 7c). Equipment for reading fluorescent signals is more common and thus cheaper, and single wavelength excitation and emission can further reduce technological requirements. However, fluorescence signals are sensitive to intrinsic and extrinsic conditions, such as salinity, pH, solvation environment of the dye, and so on. Commonly used materials are silica [101], quantum dots [109] and carbon dots [91] and upconverting NPs (UCNPs) [102].

NPs have size and shape dependent optical absorption spectra, which means that NPs of different colors can be used as immunoprobes. Using silver NPs of different colors can enable multiplexing in a single lane [21,54]. NPs of different colors are conjugated to antibodies for different biomarkers, result in different colored test lines depending on the antigen present, which was demonstrated for differentiation between dengue, yellow fever, and Ebola biomarkers (Figure 7a). The multicolor properties of NPs has also enabled the use of “barcode” particles [110] which uses NPs of different colors at predefined ratios to enable even greater multiplexing.

**SERS**. Surface enhanced Raman spectroscopy (SERS) is a technique where the Raman signal of a reporter molecule is amplified by the presence of a metal NP effectively reducing their LOD. Although a spectrometer or microscope is required for test readout, there are a number of advantages of this methodology. This lowers assay LOD [27,82,103,104] and enables multiplexing capabilities for multiple targets by simply changing the reporter molecule [22,111]. The “nanotag” format has been useful for biological detection, where a small molecule reporter is conjugated to a gold NP, and the NP linked to an antibody specific to a target. For a multiplexed Zika and dengue test, SERS can improve sensitivity to reach LODs as low as 0.72 ng/mL for Zika and 7.67 ng/mL for dengue NS1, which are physiological levels for Zika NS1.

**Electrochemical**. Electrochemical impedance spectroscopy (EIS) is a label free methodology with a relatively low sensitivity where the detection molecule is bound to a conducting surface. Concentration of the target molecule is read as the difference between the system voltage phasor and the measured phasor [105,106]. Ge et. al. proposed an electrochemical sensor where they wax printed a 6 x 4 grid, where each of the detection sites were functionalized with chitosan, multiwalled carbon nanotubes and bovine serum albumin (BSA) prior to antibody printing. After antigen binding they used a functionalized multi walled carbon nanotube to amplify the observed change in signal [37]. 

**Thermal contrast**. The photothermal properties of gold NPs can also be used to enhance the test line, where excitation of the test line with a laser and a thermal camera readout results in higher contrast relative to the background. This methodology takes advantage of the ability of metal NPs to interact with light to generate heat. Qin et. al. were able to improve LOD of an LFA by irradiating the sample with a laser (λ = 532 nm) and measuring the signal using an infrared camera [107]. Zhan et. al. used thermal contrast to improve test LOD expand test readability to approximately 6 log_10_ concentration range (Figure 7b) [108].

#### 5.1.3. Application of External Readout or Quantification Devices 

External devices or readout aids are applied to paper-based assays if the readout methodology is not visual, e.g., fluorescent, to improve the sensitivity and selectivity or to quantify the result. Such rigs can often employ low-cost, rugged hardware and leverage mobile phone hardware, combined with new software to facilitate the development of affordable, standalone instruments that can provide automated and quantitative measurements of the assays [112,113]. Integrated GPS systems can tag data with geolocation and time to enable mapping of results, which would help centralized tracking of diseases and outbreaks [114]. For example, mobile phones have been used to quantify the results of paper-based assays to detect bioagents in full blood [115], mercury in tap water [116], thyroid stimulating hormone [117], vitamin D quantification [118], toxins in agriculture [28], among many others [45,119].

## 6. Challenges of Paper-Based Assays

The core requirement for paper-based assay functionality is to have a readable signal. Beyond the ability to bind the target molecule, which is assumed for the purposes of this section, the challenges that paper-based assay technology faces are related to test sensitivity and specificity. An ideal test would have high sensitivity, i.e., be able to yield signal in response to physiological concentrations of the target molecule, while not producing a signal for via aggregation, physical adsorption, cross reactivity or other factors. This can be translated to sufficient immunoprobes successfully binding to the printed capture molecule in a specific recognition event, generating a signal. 

**Figure 8:**
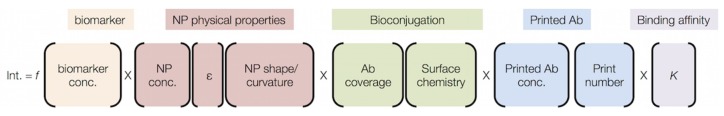
Factors influencing the test line intensity in paper-based assays. From Reference [36].

We have described the test line intensity semi-quantitatively as a function of multiple parameters (Figure 8) [120]. The signal depends on (i) the biomarker concentration; (ii) physical properties of the nanoparticle, which includes its concentration, extinction coefficient ε; (iii) the ability of the immunoprobe to bind the ligand as it relates to its surface chemistry; (iv) the amount of immobilized antibody; and (v) the target-detector affinity *K* [36,108]. 

### 6.1. Challenges Associated with the Biological Reagents

Another challenge is that the antibodies, proteins, peptides, nucleic acids which act as binding species for the target can exhibit cross-reactivity for similar biomarkers. This can result in false positives for a closely related disease (i.e., a patient infected with Zika results in a positive test for dengue). This can be a difficult issue to solve if the biomarkers are similar, such as for the NS1 for different flaviviruses. Increased stringency for antibody screening can be used to decrease cross-reactivity.

**Figure 9:**
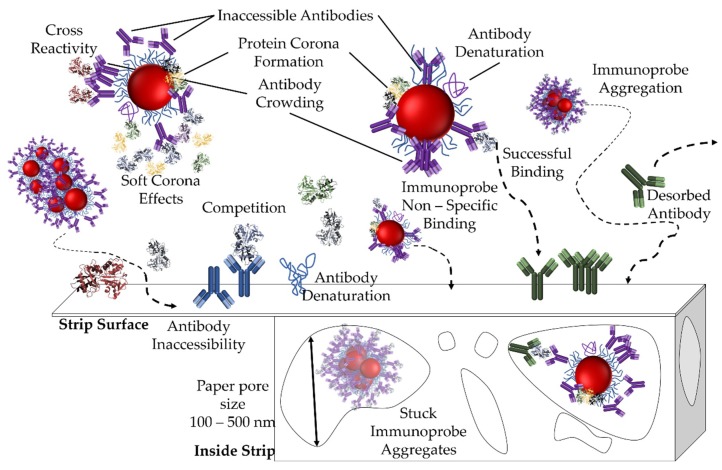
Challenges associated with paper-based immunoassays. Adapted from Reference [36]. Arrows represent particle trajectory.

On the other hand target molecules not associated with the immunoprobe can compete with immunoprobe bound antigen for the printed antibody, thus reducing the signal or producing a false negative. A similar effect may be observed due to cross-reactivity of the printed antibody (Figure 9). 

### 6.2. Substrate Related Challenges

While a relatively simple substrate when compared to cells, 3D cultures or tissues, hence its popularity, paper does present a number of challenges which need to be understood and considered when designing, working with or analyzing paper-based assays. As discussed earlier, the porosity, wetting angle and additives are important considerations. Others, such as substrate fragility are both of benefit and potential pitfall as paper may get indented, damaged or even torn as the tensile strength of unsupported paper strips is ~ 5 N/cm depending on thickness and porosity [53]. Some commercial papers have coats or covers to support or improve specific qualities of the material. Those, as well as the interactions between the paper and coat, need to be taken into account during strip design and preparation as they have a substantial impact on the results. 

Paper properties can also affect performance (Figure 9). The conversion of cellulose to nitrocellulose reduces paper wetting angle so molecules (e.g., PEG) and other modifications are added to improve substrate properties, such as paper wetting time and importantly adhesion of the printed detector molecule [75,121]. Paper modifications can impact the adsorption rate of any printed detection molecules [49,121,122]. Such alterations can also impact sample, antigen and immunoprobe adhesion and nonspecific binding, as well as possible denaturation [49,121,122]. Thus, knowing the amount and type of paper additives resulting from production is key to comparing paper-based tests made with substrate of different origin.

Paper properties can be used to improve strip design so that it incorporates multiple processes simultaneously. For example, Lu et. al. developed a paper-based system which can detect ferritin from full blood using a series of papers with different pore sizes. The authors found the methodology comparable in quality to standard centrifugation for blood separation [123].

### 6.3. Probe Related Challenges

While introducing NPs does simplify paper-based assay readout and widen applicability it also presents a number of challenges (Figure 9). 

**Immunoprobe interface**. Immunoprobes need to be colloidally stable in storage of different conditions of humidity, temperature and sample content to a degree which allows for reliable test results. Colloidal stability is dependent on both NP core and surface properties. As the core is typically chosen for specific physical properties, colloidal stability can be achieved through surface modifications [66,124,125,126]. In complex media, NP stability and properties are less dependent on surface charge and more on variables, including the presence, type and density of a ligand, due to molecular adsorption, and reduced Debye length [125,127,128,129,130,131,132].

It is generally accepted that the surface of nanomaterials entering into a solution will rapidly be decorated with various indigenous molecules. If the solution is biological the adsorbed molecules are referred to as the “biomolecular corona;” if the solution is environmental the term “natural organic matter” (NOM) and “NOM corona” is often used. 

Understanding the process of molecular adsorption, as well as its consequences is one of the major avenues of investigation in the field of nanotechnology [133]. The physical and chemical properties of nanomaterials (size, shape, surface chemistry) influence the properties of the corona that forms. As a result, understanding and controlling the NP surface interface, otherwise referred to as the biotic-abiotic or nano-bio interface, has large bearing on the NP material properties and behavior *in vitro* and *in vivo,* including its ability to bind antigens in paper-based assays [36,66,134,135].

Protein coronas and nano-bio interfaces have been studied extensively for when NPs are used as drug delivery vehicles or imaging agents for cancer. However, these interface issues are also present in paper-based assays, as NPs are also in a complex biological fluid when the samples are run (Figure 9). Arguably, the complexity of the interface is greater in paper-based assays. The grafted antibody can denature, be oriented incorrectly for antigen binding [136,137], or be obscured by the NP surface ligand. The biological fluid is under flow in a highly obstructed medium with pore size comparable to that of the immunoprobe, as it wicks through the strip antibody-antigen binding is not at equilibrium. This could lead to probe aggregation, non-specific adsorption, and steric hindrance of the antibody on the NP surface. All of these interface issues can detract from assay function, compromising its specificity and sensitivity and cause unwanted side effects, such as poor signal to noise. 

In a diagnostic, non-specific adsorption is critical, because it can result in false positives or negatives, which can have serious consequences. For example, a false positive for Ebola virus would mean that the patient could be quarantined with other infected Ebola patients, because of its facile human-to-human transmission. 

Surface ligands are often used to mitigate interactions on the nano-bio interface and impart desirable properties, such as resistance to protein binding [124,138,139]. Furthermore, the functionality of surface bound detector molecules has previously been linked to the properties of ligands under or around them [126,128,138]. However, surface ligands may also sterically hinder the antibody on the NP surface of the immunoprobe, reducing its ability to bind to the antigen.

### 6.4. Environment Related Challenges

Environmental factors have the potential to severely impact the commercial applicability of paper-based assays. The environmental conditions at which the diagnostics are run can often differ significantly from laboratory conditions. Tropical areas have higher temperatures and humidity which can compromise test stability. If the antibodies in the test are not stabilized, then may denature under these conditions (Figure 9). To address this, packaging can help stabilize the device, or stabilizers can be added to the tests to improve the stability of the biologics.

### 6.5. Sample Related Challenges

All of the challenges listed above are related to the probability of the immunoprobe to bind the target molecule in the scenario of a real patient or field sample. It is increased by the number of functional detector molecules on a structurally and colloidally stable immunoprobe and reduced by crosslinking and interference by other molecules. To this end, several sample-related factors need to be considered during test design.

**Type of sample**. One important factor is the sample type, e.g. blood or urine, due to the varying concentration of some target molecules and sample conditions. General protein content and type, salinity and pH vary in different sample types which may need to be accounted for in the immunoprobe design. Target biomarkers can have different concentration ranges in different sample types (i.e., high in blood but low in saliva).

**Patient-to-patient sample variability**. Factors, such as age, sex and medical history, would impact test results. As mentioned above, detection of anti-OspA may be the result of previous vaccination or infection as IgGs can persist months and years after the infection is cleared [58]. 

**Sample variability**. Sample content may vary for the same patient due to diet, time and content of last meal, alcohol or medication intake, disease cycle, immune response, etc. For example, immune response to an infection is time dependent, in early stages IgM levels for the antigen are high, but decrease with time. IgG production starts later, but may persist for a long time. 

**Sample handling**. Assay results are also influenced by sample handling, storage, and testing conditions, as these influence not only the structure of the target molecule but also the test behavior (flow rate, etc.). The time of testing, sample storage, time outside of storage prior to testing (to allow for temperature equilibration for example) and time of incubation with the immunoprobe may also be relevant (Figure 9). 

As sample related challenges are highly complex, it can be useful to consider them first. For example, in a test for an infectious disease the first step would be to consider its cycle as this would key to finding targets to detect, the type of sample they are in, concentration and state. This would be crucial for immunoprobe and test design and initial development stages. 

## 7. Conclusions and Future Prospects

There are many exciting future directions for paper-based assays. Infectious diseases are spreading and occurring with a higher prevalence, leading to projected increases in burdens for many diseases. Consequently, it is anticipated that there will be a greater need for rapid diagnostics for new and emerging outbreaks. Even as vaccines and drugs are developed, the need for a diagnostic still remains for proper patient treatment.

In addition, paper-based assays have many potential application spaces beyond infectious disease, including drug testing, food safety, counterfeit detection, and many others. Indeed the possibility of coupling simple, user friendly devices with state-of-the-art communication technology (such as smart phones) could allow for real time monitoring of disease outbreaks, as well as lower the cost and simplify long term monitoring of public health concerns, e.g., blood cholesterol [140]. Outside of healthcare such technology would help governments monitor and control food pathogens, harmful substances [141], and regulatory adherence. Thus, further developments and wide scale deployment of POC devices can substantially change how we understand healthcare, agricultural and environmental regulation, harmful substance control among others. 

While the operation of paper-based assays are simple, their operational details are multifaceted. The applicability of paper-based assays can be determined by parameters starting from the direction of the paper cutter during manufacturing and the amount of water used during the pulping process to the details of the nano-bio interface on the immunoprobe and how it interacts with environmental components, as well as the running conditions of the test. Furthermore, there are multiple strategies which can enhance the ability of these tests to successfully detect a target molecule, such as improving probe stability or using signal enhancement. This wide dependence can result in much of the assay development process being *ad hoc* which has been a successful in -lab strategy allowing for the quick development of tests for hundreds of target molecules. However, it is unlikely that this success can be translated into a large-scale R&D production of commercially viable devices without at least a partial understanding of a broad enough range of the above parameters. Furthermore, strategic use of such data may allow for advanced designs which can relatively easily and cheaply conduct a multistep analysis in a single device. 

## Figures and Tables

**Table 1:** Definition of sensitivity and specificity.

**Sensitivity [73]** Probability of Detecting the Target Molecule when Present		**Specificity [73]** Probability of not Detecting the Target Molecule when Absent	
$\mathrm{Sensitivity}=\frac{N_{\mathrm{True}\;\mathrm{Positives}}}{N_{\mathrm{True}\;\mathrm{Positives}}+\;N_{\mathrm{False}\;\mathrm{Negatives}}}$	(4)	$\mathrm{Specificity}=\frac{N_{\mathrm{True}\;\mathrm{Negative}}}{N_{\mathrm{True}\;\mathrm{Negative}}+\;N_{\mathrm{False}\;\mathrm{Positive}}}$	(5)
$N_{\mathrm{True}\;\mathrm{Positives}}$: The number of times the test detected the target molecule in a sample when it was present. $N_{\mathrm{False}\;\mathrm{Negatives}}$: The number of times the test failed to detect the target molecule when it was present.		$N_{\mathrm{True}\;\mathrm{Negative}}$: The number of times the test did not detect the target molecule in a sample when it was not present. $N_{\mathrm{False}\;\mathrm{Positive}}$: The number of times the test detected the target molecule when it was not present.	

**Table 2:** Novel readout strategies applied in paper based immunoassays.

Classification	LOD	Linear Range	Advantages	Limitations	References
Optical readout	ng/mL range	ng/mL to ug/mL	Quick Single step run Inexpensive Easy to operate anywhere	Low sensitivity for certain applications	[82]
Optical enhancers					
- Isotachophoresis	60–400 fold improvement	0.1–10 mg/L	Improved sensitivity Easy to operate	Signal depletion over time	[94]
- NP aggregation	1000 fold improvement	0.5 pM–50 nM		Additional operation step, less robust to climate conditions	[44]
- secondary probe	51 (96), 100 (95), 1000 (97) fold improvement	0.01–30 ng/mL		Additional binding event	[95,96,97]
- probe growth	100-fold (99,100)	10^10^–10^13^ RNA copies		Less control in probe growth between samples	[98,99,100]
Fluorescence readout	ng-pg/mL range	ng/mL to 10 μg/mL	Up to 1000-fold improvement (94)	Special equipment required, access to electricity	[94,101,102]
SERS	ng/mL (81,105)	1–100 ng/mL, 10^2^–10^6^ cfu/mL	Up to 10000-fold improvement (105)	Expensive equipment and trained personnel	[82,103,104]
Electrochemical	ng-μg/mL	0.1–1.0 ng/mL			[105,106]
Thermal contrast	nM range	10^−7^ to 10^−2^ titer			[107,108]
